# Monolysocardiolipin (MLCL) interactions with mitochondrial membrane proteins

**DOI:** 10.1042/BST20190932

**Published:** 2020-05-26

**Authors:** Anna L. Duncan

**Affiliations:** Department of Biochemistry, University of Oxford, Oxford, U.K.

**Keywords:** cardiolipin, mitochondria, monolysocardiolipin, protein–lipid interactions

## Abstract

Monolysocardiolipin (MLCL) is a three-tailed variant of cardiolipin (CL), the signature lipid of mitochondria. MLCL is not normally found in healthy tissue but accumulates in mitochondria of people with Barth syndrome (BTHS), with an overall increase in the MLCL:CL ratio. The reason for MLCL accumulation remains to be fully understood. The effect of MLCL build-up and decreased CL content in causing the characteristics of BTHS are also unclear. In both cases, an understanding of the nature of MLCL interaction with mitochondrial proteins will be key. Recent work has shown that MLCL associates less tightly than CL with proteins in the mitochondrial inner membrane, suggesting that MLCL accumulation is a result of CL degradation, and that the lack of MLCL–protein interactions compromises the stability of the protein-dense mitochondrial inner membrane, leading to a decrease in optimal respiration. There is some data on MLCL–protein interactions for proteins involved in the respiratory chain and in apoptosis, but there remains much to be understood regarding the nature of MLCL–protein interactions. Recent developments in structural, analytical and computational approaches mean that these investigations are now possible. Such an understanding will be key to further insights into how MLCL accumulation impacts mitochondrial membranes. In turn, these insights will help to support the development of therapies for people with BTHS and give a broader understanding of other diseases involving defective CL content.

## Introduction

Monolysocardiolipin (MLCL) is a three-tailed glycerol-phospholipid and an intermediate product in the biosynthesis and degradation of cardiolipin (CL) [1]. In eukaryotes, CL is found uniquely in mitochondrial membranes, comprising 10–20% of the lipid content of the inner mitochondrial membrane [2]. CL is also found in bacteria, reflecting the common evolutionary origins of mitochondria and bacteria [3]. MLCL accumulates in the mitochondrial membranes of people with Barth syndrome (BTHS) [4], a genetic disease caused by mutations in a transacylase enzyme, tafazzin, involved in CL remodelling.

CL is involved in many mitochondrial processes: CL has a crucial role in mitochondrial energy production, interacting with and enhancing the activity of all the major respiratory chain proteins [5] as well as roles in cristae morphology [6], apoptosis [7], mitophagy [8], and mitochondrial fusion and fission [9,10]. It is, therefore, not surprising that the deficiency of tafazzin, resulting in the accumulation of MLCL and other CL variants, and a decrease in total CL content, might cause problems [11]. However, there remains much to be understood regarding the molecular mechanisms underlying the effect of the change in lipid content and its impact in disease [11–13].

Here, the reason for MLCL accumulation, the effect of this build-up in mitochondrial membranes, and what we know about the protein–MLCL interactions involved are reviewed.

## The structure and physical properties of MLCL

**Figure 1.**
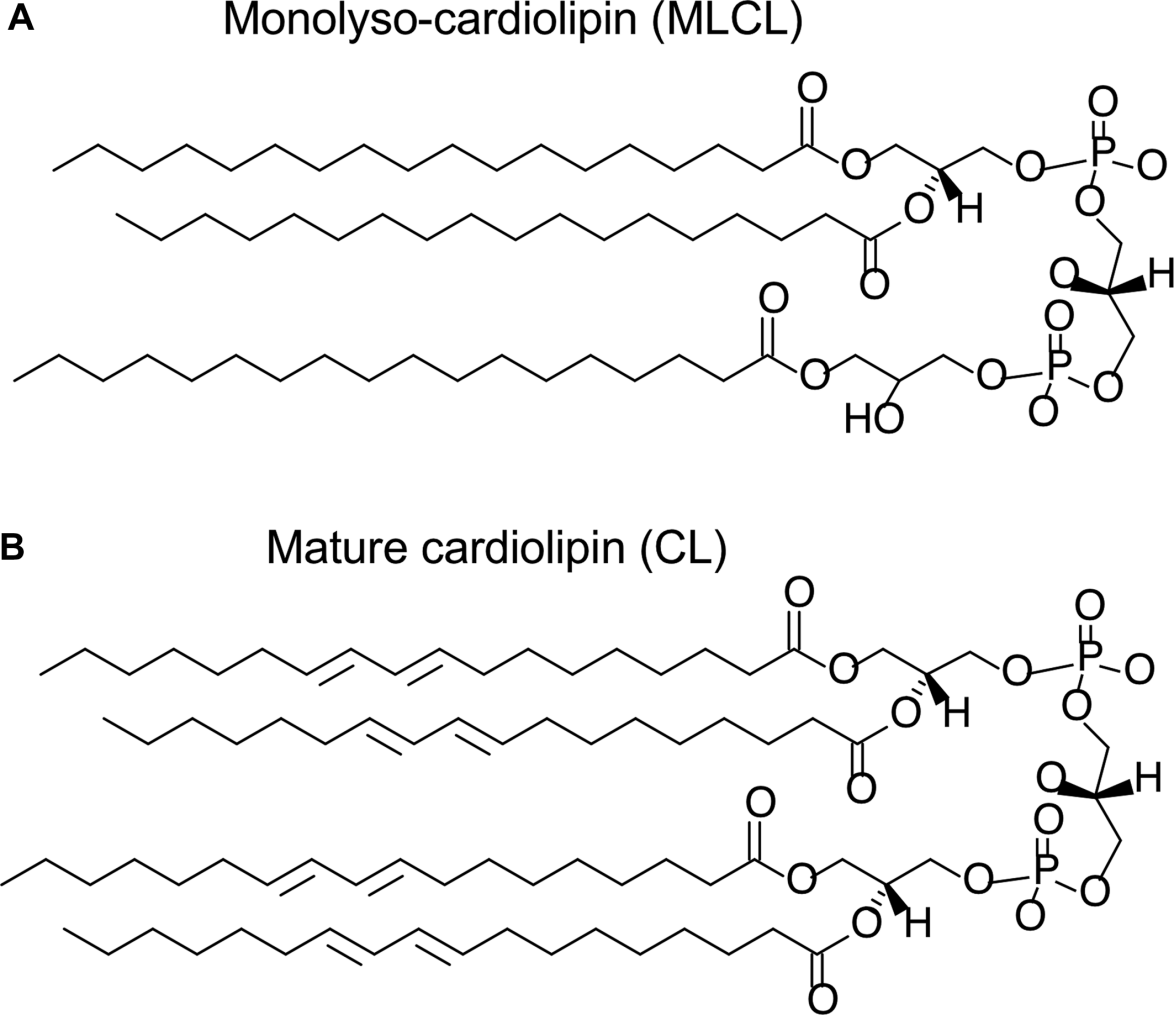
Chemical structure of MLCL (**A**) and mature, tetralinoleoyl-CL (**B**).

The chemical structure of MLCL and the mature (remodelled) form of CL is shown in Figure 1. The distinctive features of CL, as compared with other lipids found in eukaryotic cells, are its geometric and electrostatic properties: CL has a conical shape due to its small headgroup and four acyl chains, and its two phosphate groups confer a double negative charge. These properties help to inform an understanding of the physical properties of MLCL.

### MLCL, CL and membrane curvature

Due to its geometry, CL has been shown *in vitro* to be sorted to membranes with negative curvature [14]. Molecular dynamics simulations of CL-containing membranes also show localisation of CL to curved regions of the membrane [15–17]. MLCL, on the other hand, has one less acyl chain, therefore, would be expected to have less of a propensity to localise to curved membrane regions, indeed MLCL-containing membranes have been shown to have a greater preference for a lamellar phase than CL [18]. Molecular dynamics simulations have also shown that MLCL does not localise specifically to curved regions of the bilayer [19] and that MLCL bilayers take on less negative curvature than bilayers containing CL [20].

### MLCL and CL headgroup charge

There has been some controversy over the charge of CL at physiological pH. CL has been shown to contain only one negative charge in bulk bilayers at physiological pH, rationalised by assuming that the headgroup ‘traps’ one proton on one of the phosphate moieties by forming a tight bicyclic H-bonding structure with the oxygen from the connecting glycerol moiety [21]. The finding led to the proposal that CL acts as a proton shuttle [22]. However, subsequent work has since shown that CL exists with a double negative charge at physiological pH [23–25]. Furthermore, the putative bicyclic H-bonding network has a particular headgroup geometry; however, this is not observed in crystal structures of protein–CL complexes [26]. It has been demonstrated *in silico* that MLCL has a double negative charge at physiological pH [24].

### MLCL vs. CL headgroup geometry

All-atom molecular dynamics simulations comparing MLCL and CL behaviour in bilayers suggest that the hydroxyl group on the lyso side of the MLCL headgroup (Figure 1A) is prone to orient itself more towards the interfacial polar region than the hydrophobic core of the membrane, causing the lyso side of MLCL to be ‘pulled’ towards the solvent phase and leading to headgroup tilt, whereas the headgroup of CL displayed no such tilting [19]. The tilting of the MLCL headgroup means that the phosphate on the lyso side of MLCL is also positioned slightly further from the bilayer hydrophobic core than either phosphate moiety in the CL headgroup. This has possible implications for MLCL–protein interactions as compared with CL–protein interactions, given that the two phosphates on CL often form ionic interactions with positively charged protein side-chains [27]. Molecular dynamics simulations also showed that the acyl chain on the lyso side of MLCL was more ordered than other acyl chains [19].

### MLCL and CL acyl chain content

The acyl chain content of MLCL in BTHS cells has a low degree of unsaturation [28,29] whereas remodelled, mature CL has a higher level of unsaturation, although this does vary between tissue types [30]: (18:2)_4_ acyl chain content is particularly tightly controlled in mammalian cardiac tissue [31], while brain CL is more diverse [32–34].

## How does MLCL accumulate?

**Figure 2.**
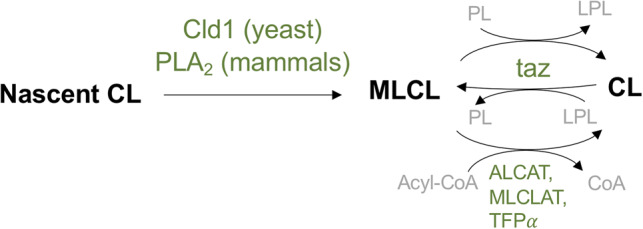
Schematic of CL remodelling. Nascent CL is deacylated by Cld1 in yeast; phospholipase A_2_ (PLA_2_) in mammals. Tafazzin transfers an acyl chain from another phospholipid (PL) to MLCL, forming CL and a lysophospholipid (LPL). After one or more cycles, this will result in the mature form of CL. CL acyltransferase enzymes, which take MLCL and acyl-CoA as substrate, are also shown: acyl-CoA:lysocardolipin actyltransferase 1 (ALCAT), monolysocardiolipin acyltransferase 1 (MLCLAT), and trifunctional protein alpha subunit (TFPα). Enzyme names are coloured green and lipid names are in black.

MLCL is produced from CL by a lipase removing a single acyl chain from CL, which can occur as part of CL remodelling or degradation. To understand the accumulation of MLCL, it is first useful to briefly review CL biosynthesis and remodelling. The biosynthesis of nascent CL occurs in the mitochondrial inner membrane from phosphatidylglycerol (PG) and CDP-diacylglycerol [35]. The acyl chain composition of nascent CL is remodelled, that is, acyl chains are removed and replaced, to form the mature form of CL, which typically has higher unsaturated content. In yeast, CL deacylation is performed by a CL-specific phospholipase Cld1, which has a specificity for saturated tails [36,37], whereas in mammals, phospholipase A_2_ (PLA_2_) is responsible [38–40], most likely the calcium-independent iPLA_2_ gamma *in vivo* [41]. Tafazzin can then reacylate MLCL [42] (Figure 2). Tafazzin uses acyl chains from other phospholipids to reacylate MLCL, or to act as a CL-transacylase. The specificity of tafazzin remains controversial: it has been demonstrated that tafazzin may have some acyl specificity [43]; however, the work from Schlame *et al.* [44] shows that the specificity of tafazzin is dependent on the physical properties of the bilayer, particularly the ability of the bilayer to form non-bilayer phases. There are other remodelling enzymes: CL can also be reacylated by acyl-CoA:lysocardolipin actyltransferase 1 (or ALCAT1) in mitochondrial-associated membranes [45] and in mitochondrial membranes by monolysocardiolipin acyltransferase 1 (or MLCLAT1) [46]. There is also evidence that the trifunctional protein alpha subunit, which is similar to MLCLAT1, is involved in CL remodelling [47–49].

MLCL accumulation occurs in people with BTHS leading to an increase in the MLCL:CL ratio, and otherwise, MLCL is not present in normal tissue, except for testis [50]. The cause of an increase in the MLCL:CL ratio in BTHS is yet to be fully understood [1]. Initially, MLCL accumulation was thought to be due to tafazzin being part of a deacylation–reacylation cycle, with tafazzin deficiency causing the remodelling process to stall, meaning that MLCL was not being reacylated. However, more recently, it has been shown that MLCL accumulation may occur due to MLCL being an intermediate in CL degradation [51]. This hypothesis is based on the finding that tafazzin deficiency in yeast increases CL turnover, while knock-down of Cld1 decreases CL turnover. The rationale is that due to less favourable interaction of MLCL and saturated CL within complexes of mitochondrial membrane proteins, saturated CL is exposed to degradation to MLCL, which creates a cycle culminating in the accumulation of MLCL.

## MLCL accumulation in disease

MLCL accumulates in people with BTHS [4,52,53], leading to the characteristic increased MLCL:CL ratio. MLCL has also been shown to build-up after cardiac arrest in rats [54], and the lack of CL remodelling has also recently been associated with cardiac arrhythmia [47]. However, despite defective or decreased CL content being implicated in many other disease states (e.g. cancer, neurodegenerative disease, and diabetes), the accumulation of MLCL is not widely found in other CL-linked diseases [11]. As introduced above, BTHS is known to be caused by a mutation in the tafazzin gene [55].

People with BTHS present with myopathy, heart failure, muscle weakness, growth retardation and neutropenia [56]. On a cellular and molecular level, cells from people with BTHS have a reduced mitochondrial membrane potential [57], lower electron transport chain protein activity [58,59], decreased respiratory coupling index [60] and increased proton leak [29] (leading to possible further mitochondrial damage via the increased production of reactive oxygen species [29,60]), destabilised respiratory supercomplexes [61], and abnormal cristae structures [62]. Mutations in taffazin also cause altered ATP synthase organisation in *Drosophila melanogaster* [63].

As well as a lower concentration of CL and altered CL composition (i.e. a higher proportion of CL with saturated tails), an increase in the MLCL:CL ratio is a key marker for BTHS [64,65]: although dependent on cell type, the MLCL:CL ratio typically increases from a range of 0.0–0.2 in control cells to the significantly different range of 0.4–100 in cells from BTHS patients [28,64,66].

The reasons for altered lipid content causing BTHS symptoms, and the functional role of tafazzin remains an open question, although the existence of BTHS underlines its importance [1,11,13,35]. Recently, it has been suggested that the main role of tafazzin is to remodel CL such that it lowers the energetic cost of protein crowding in the protein-dense inner mitochondrial membrane: Xu *et al*. [51] showed that MLCL associates less tightly with mitochondrial proteins and that the association of CL in respiratory supercomplexes protects CL from degradation. The authors also showed that CL unsaturation promotes protein association, which in turn is thought to protect CL from degradation. In further work, Xu *et al.* [37] showed that the expression of complexes of the respiratory chain triggers remodelling; knock-down of other mitochondrial proteins, even including others that inhibit the respiratory function, did not alter CL composition. This lead to the suggestion that tafazzin remodels CL to have unsaturated tails since these will lower the energetic cost of the membrane packing around respiratory complexes in the inner mitochondrial membrane. It was argued that as a result of this, respiratory complexes can be incorporated into the mitochondrial membrane with higher density, without loss of membrane stability. This might explain why membranes with high energy requirements are most vulnerable to tafazzin deficiency [56].

## The nature of MLCL interactions with mitochondrial proteins

The result that MLCL associates less tightly than CL with mitochondrial membrane proteins [51] forms a key part of understanding how the MLCL:CL ratio increases in people with BTHS and why MLCL accumulation might affect CL remodelling. It also provides a rationale for the destabilised respiratory supercomplexes and thereby reduced membrane potential and lower electron transport chain protein activity [58,59] found in people with BTHS. The weaker association of MLCL vs. CL was demonstrated using ^31^P-NMR spectroscopy, which showed that MLCL was solubilised by digitonin, a relatively weak detergent, whereas CL and MLCL were solubilised by SDS (sodium dodecyl sulphate), a stronger detergent [51]. The nature of MLCL–protein interactions remains unclear but is a vital part of the picture.

### MLCL interaction with proteins involved in oxidative phosphorylation

CL is known to interact with, and affect the activity of, all of the key protein complexes of oxidative phosphorylation, namely: Complex I [67,68], cytochrome *bc_1_* (Complex III) [69], cytochrome *c* oxidase (Complex IV) [70] and ATP synthase [71–73] as well as the ADP/ATP carrier [74], the mitochondrial transporter responsible for shuttling newly synthesised ATP out of the mitochondrial matrix towards the rest of the cell.

In contrast, binding of MLCL to cytochrome *c* oxidase occurs but with lower affinity than CL. MLCL retained 60% of the activity of cytochrome *c* oxidase compared with CL, and when CL is depleted altogether, the enzyme has only 30–50% of the original activity [75]. This agrees with the findings of Xu *et al.* [51] that MLCL interacts less tightly than CL, and further suggests that the loss of the activity of cytochrome *c* oxidase reconstituted with MLCL rather than CL is related to lower MLCL affinity. The partial retention of activity with MLCL indicates that MLCL is in some ways able to mimic CL interaction.

The ADP/ATP carrier was also reported to retain only 12% of activity compared with CL on reconstitution with MLCL [76]. In this case, it is not necessarily clear whether the loss of activity with MLCL compared with CL is due to the loss of association or whether the MLCL could be as tightly bound, but with a lesser impact on activity; compounding the difficulty in teasing these apart is the lack of knowledge of how exactly CL optimises the activity of this carrier.

### MLCL and proteins involved in apoptosis

In cultured lymphoblast cells from people with BTHS, apoptosis was found not to be increased [4]. However, MLCL interactions with many proteins involved in apoptosis have been investigated demonstrating that MLCL interacts with tBid more tightly than CL [77,78], caspase-8 [29] less tightly than CL, and also modifies the release of cytochrome *c* [78,79].

Bid is a proapoptotic protein and member of the Bcl-2 family. Bid is cleaved by caspase-8 to form tBid, which then associates at the mitochondrial outer membrane, leading to oligomerisation of other Bcl-2 family proteins, Bax and Bak, and the release of cytochrome *c* [80]. CL had been suggested to target tBid to the OMM during apoptosis [81]. Using electron spray mass spectroscopy, MLCL was shown to enhance Bid and tBid mitochondrial membrane association, relative to CL [78]. MLCL also appeared to enhance the release of cytochrome *c* [78] and affect the oxidation state of cytochrome *c* [79]. Furthermore, MLCL was shown to be capable of inducing tBid dissociation from nBid (the other Bid-cleavage product) [77]. Although there exists structural information regarding tBid [82–84], the nature of the interaction with CL and MLCL is not clear [77]. In contrast with Bid, MLCL binds less tightly to active caspase-8 than CL [29]. Thus, whether apoptosis is be enhanced or otherwise by MLCL accumulation remains a somewhat confounding picture.

The enhanced interaction of Bid with MLCL vs. CL is intriguing, as it contrasts with the findings showing that MLCL associates less tightly with mitochondrial proteins [51]. However, Bid is a membrane-associated rather than a membrane-embedded protein.

### What does this tell us about MLCL–protein interactions?

It is not clear why the loss of an acyl chain in MLCL causes the loss of interaction with proteins and the loss of activity compared with CL. A computational study of MLCL properties [19], discussed above, showed that due to the loss of an acyl chain, the lyso side of the MLCL headgroup was tilted away from the hydrophobic core, and the acyl chain on the lyso side of MLCL was more ordered. Both of these differences may cause a weakening of the association with cytochrome *c* oxidase and possibly the ADP/ATP carrier, while retaining some of the characters of the CL interaction. The ordering of the lyso acyl chain in MLCL may also contribute to the preference for MLCL not to be sequestered in respiratory supercomplexes [37,51]. The fact that non-embedded membrane protein Bid interacted more tightly with MLCL than CL may also be due to a headgroup tilt that exposes the lyso phosphate oxygen to the solvent phase.

EPR studies of the Na^+^/K^+^ ATPase show that CL and MLCL have a similar affinity for interaction with the protein [85], which could indicate that for any difference to be observed, there would have to be a tightly interacting CL binding site; since there is no evidence for the existence of a mitochondrial Na^+^/K^+^ ATPase [86], it might be assumed that the protein has not evolved to have optimal CL binding. It may be in this case, therefore, that the rearrangements in the MLCL headgroup are too subtle to make a difference.

However, without further studies, it is not certain that the MLCL headgroup rearrangements observed in bulk in bilayers [19] will translate to alterations in protein–lipid interactions.

Confounding any interpretation of MLCL–protein interactions is the problem that the molecular mechanism through which CL optimises protein activity is itself unclear. However, this does mean that a greater understanding of MLCL–protein interactions may also help to shed light on the role of CL.

## Conclusions and outlook

A key aspect of understanding the reasons for MLCL accumulation and the effect of MLCL accumulation in mitochondrial membranes lies in understanding MLCL interaction with mitochondrial proteins. Recent work has shown that MLCL interacts less tightly with mitochondrial membrane proteins [51] and that global impairment of respiratory chain complexes triggers MLCL accumulation [37], leading to the hypothesis that an increase in the MLCL:CL ratio impacts the stability of mitochondrial membranes, in particular impairing the tight-packing of the respiratory supercomplexes. To test this hypothesis, it will be important to understand more about the nature of MLCL–protein interactions. Existing data indicate that the loss of an acyl chain in MLCL diminishes membrane-embedded protein association at high-affinity CL interaction sites [75,76], possibly due to slight rearrangement in the MLCL headgroup as compared with CL [19]. MLCL association with peripheral membrane proteins does not necessarily follow the trend [78]. However, it remains unclear how MLCL interacts with mitochondrial membrane proteins and why it does so less tightly than CL with mitochondrial membrane-embedded proteins.

### Other possible effects of MLCL accumulation

Altered mitochondrial morphology, i.e. deformed cristae, is a classic feature of BTHS. In a fly BTHS model, ATP synthase organisation is altered, which may influence cristae structure, since it has been shown that rows of ATP synthase dimers form on cristae edges, which drives membrane curvature [87–90]. Given that CL interacts directly with ATP synthase, albeit transiently [91], and that CL is known to localise to curved regions, whereas MLCL does not [19], it is conceivable that differences in MLCL interaction with ATP synthase may contribute to altered mitochondrial morphology in BTHS. However, it could also be that other CL-associated cristae-organising machinery [92] are also affected by abnormal CL composition, which then impacts on ATP synthase organisation. MLCL interaction with ATP synthase dimers and even the localisation of MLCL within cristae structures *in vivo* is unclear.

More generally, it is also possible that the accumulation of MLCL may disrupt mitochondrial membrane properties, leading to further effects on the mitochondrial protein function. MLCL-containing bilayers, as mentioned above, are more likely to be flat [18–20] and are less susceptible to shape deformation under pressure than CL-containing bilayers [19]. Since the mitochondrial inner membrane is highly curved, there may be many proteins, localised to cristae tips, as discussed above, or at cristae junctions, or which perform dynamic functions during mitochondrial fusion and fission, that therefore could be affected by an increase in the MLCL:CL ratio. MLCL-containing bilayers are also less likely to have small ‘defects’, where the hydrophobic core of the bilayer is exposed [19]. This may mean that, for peripheral proteins where partial interaction of the protein with the hydrophobic core of the membrane is the mode of binding, MLCL-containing bilayers form less favourable interactions.

### Advances in investigating protein–lipid interactions

Although there remains much to be understood about the role of MLCL and its interaction with mitochondrial proteins, we are currently at an exciting time for research into protein–lipid interactions, since advances in both experimental and computational methods promise (and already are starting to achieve) significant advances.

While there have been some notable cases of CL being resolved directly in crystal structures (e.g. the ADP/ATP carrier [74,93], cytochrome *bc*1 [94], and cytochrome *c* oxidase [95]), the cryo-electron microscopy ‘resolution revolution’ [96] means that there are now many more membrane protein structures with CL resolved, such as the yeast respiratory supercomplexes [97,98], mammalian Complex I [99,100], and ATP synthase [101]. As such, there are not to my knowledge any structures of mitochondrial proteins resolved in the presence of MLCL. Such structures would give invaluable insights as to the molecular detail of how MLCL interacts with mitochondrial proteins, and how that may differ from interaction with CL, although the obvious problem might be that with less tight association, MLCL is less likely to be resolved in any structures. Similar advances in cryo-electron tomography have led to visualisation of membrane proteins in intact mitochondria with unprecedented resolution [102]. However, again, to my knowledge, this technique has not been applied to mitochondria of people with BTHS, or more generally, cells with a high MLCL:CL ratio. Cryo-electron tomography would allow us to understand further how certain distinctive mitochondrial proteins (such as Complex I, ATP synthase, and cytochrome *bc*1) are organised in the disrupted cristae of BTHS mitochondria, and in particular, how the arrangement of respiratory supercomplexes is altered.

Mass spectroscopy has, as discussed above [37,51], provided invaluable insight into MLCL–protein interactions. New techniques that allow for mass spectroscopy of proteins ejected directly from native mitochondrial membranes have also recently been published [103]. Although such an approach may require some fine tuning [104,105], this methodology could potentially be used to look at the mitochondria of people with BTHS and would allow further understanding of the extent of MLCL–protein interaction and effects of MLCL accumulation [106].

**Figure 3.**
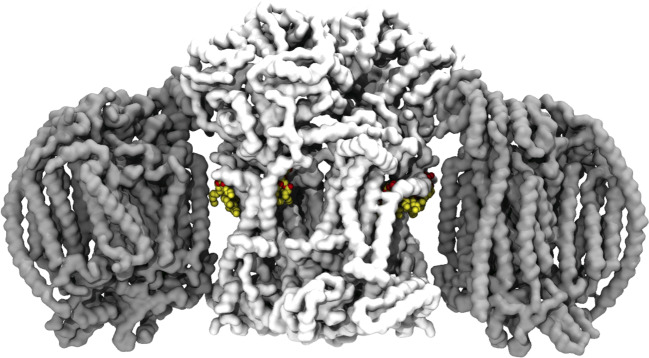
Cryo-electron microscopy structure of a yeast supercomplex. A yeast supercomplex (PDB ID: 6HUY [98]) containing a dimer of cytochrome *bc*1 (or Complex III; in white), two monomers of cytochrome *c* oxidase (Complex IV; grey), with resolved cardiolipin lying in the protein–protein interfaces (cardiolipin is shown as spheres: carbon atoms in yellow; oxygen, red; phosphorous, brown).

Computational techniques, in particular molecular dynamics simulations, have also seen significant development [107]. Molecular dynamics simulations allow multiple lipid interaction sites to be identified and characterised [108,109], and new methodologies mean that relative free energies of lipid binding can be assessed [110]. Molecular dynamics simulations have been used successfully to investigate CL interactions with mitochondrial [111–115] and bacterial [116–118] proteins, although, to my knowledge, have yet to be extended to investigate MLCL. Simulations of MLCL embedded in mitochondrial membranes containing mitochondrial proteins would demonstrate differences between interaction sites of MLCL and CL and enable the calculation of MLCL vs. CL binding free energies. Coarse-grained molecular dynamics simulations [119] of crowded mitochondrial membranes (e.g. [120,121]) will further help to show how protein crowding impacts on MLCL–protein interactions. We now have a good indication of where CL would bind in yeast supercomplexes [97,98] (Figure 3). Given the increasing sophistication of molecular dynamics simulations and their success in reproducing the effect of membrane curvature on MLCL and CL localisation [19], it appears that the use of simulations could give an exciting insight into the hypothesis that MLCL is less favourably sequestered than CL in respiratory supercomplexes, and the nature of MLCL–protein interactions more broadly.

Such advances will help to shed light on possible therapies both for BTHS, a disease specifically caused defective CL remodelling leading to defective CL content and accumulation of MLCL as well as the much broader category of pathologies where CL content is altered.

## Competing Interests

The author declares that there are no competing interests associated with this manuscript.

## Perspectives

- A marked increase in the MLCL:CL ratio is the hallmark of people with BTHS, a genetic disease caused solely by defective CL remodelling. People with BTHS have mitochondria displaying defective oxidative phosphorylation and abnormal cristae structure, causing multi-system disorder with potentially life-limiting effects.
- It has been shown that MLCL associates less tightly with many inner membrane proteins, and this finding underlies an explanation for how an increase in the MLCL:CL ratio affects people with BTHS. However, the nature of MLCL interaction with mitochondrial proteins is unclear.
- Structural, analytical, and computational techniques, used in combination, are poised to provide answers.

## Open Access

Open access for this article was enabled by the participation of University of Oxford in an all-inclusive *Read & Publish* pilot with Portland Press and the Biochemical Society under a transformative agreement with JISC.

## Abbreviations

BTHS: Barth syndrome
CL: cardiolipin
MLCL: monolysocardiolipin
